# Dog-Assisted Therapies and Activities in Rehabilitation of Children with Cerebral Palsy and Physical and Mental Disabilities

**DOI:** 10.3390/ijerph120505046

**Published:** 2015-05-12

**Authors:** Dilek Tunçay Elmacı, Sibel Cevizci

**Affiliations:** 1 Private Gercek ILGI Training and Rehabilitation Center, Perge Branch, Antalya 07200, Turkey; E-Mail: dlktncylmc@gmail.com; 2 School of Medicine, Public Health Department, Canakkale Onsekiz Mart University, Canakkale 17100, Turkey

**Keywords:** cerebral palsy, children, dog-assisted therapy, mental disability, physical disability

## Abstract

The aim of the present study was to evaluate dog-assisted therapies and activities in the rehabilitation of children with cerebral palsy and physical and mental disabilities who have difficulties in benefiting from well-being and health-improving services. This descriptive-explanatory study was conducted in disabled children of various ages between 2008 and 2011 by an experienced team in a private training and rehabilitation center in Antalya (Turkey). In this study, five study groups were formed among the children with physical and mental disabilities. During the therapy studies, three dogs were used. For each therapy group, the goals for the children and therapist were defined, and the activities were determined according to these goals. The entire study process was followed using audio-records and photographs of patients. The expected targets were reached in all study groups. The children who experienced fear, anxiety and difficulties due to their disabilities in daily life learned to cope with their anxieties and fears, set goals and make plans to achieve their aims. During this study, the children improved their abilities to use their bodies according to their capabilities. Accordingly, they improved their ability to develop empathy between themselves and a therapy dog, to receive and present help, and to communicate. The results of the present study revealed that dog-assisted therapies and activities can be a supportive method for routine treatment procedures in the rehabilitation of children with cerebral palsy and physical and mental disabilities.

## 1. Introduction

Dog-assisted therapy is a model that is widely used in animal-assisted therapy (AAT). AAT is a supportive, goal-oriented intervention that primarily results in human and animal interaction [1,2,3,4,5,6]. The positive-helpful bond that results from this interaction is the basis for the effect mechanism of AAT. This curative effect comprises four basic mechanisms: psychological, emotional, playing, and physical stimulation, according to Ballarini [4]. All of the mechanisms together demonstrate the psychosomatic effects of the human-animal bond and the interaction during AAT and animal-assisted activities [5,6]. Lafrance *et al.* reported that patients’ social and verbal behaviors may improve in the presence of a therapy dog [7]. Additionally, AAT can be beneficial for the rehabilitation of life quality and psycho-social behaviors [8]. Various researchers have reported that AAT should be considered when planning the treatment of individuals with physical and mental disabilities such as dementia [1,9,10,11,12].

The interaction between an animal and human results in an increase in neurochemicals, which initiates a decrease in blood pressure and induces relaxation. This relationship may be beneficial for ameliorating agitated behavior and psychological symptoms of chronic diseases that involve physical and mental disabilities. Richeson revealed that AAT can increase social interactions by decreasing the agitated behaviors of patients with dementia [12]. Kongable *et al.* observed that a therapy dog increased patients’ social behaviors, including smiling, laughing, looking, touching, and verbalizing [13].

Animals can act as transitional objects, which allows people to first establish a bond with them and then extend this bond to people. Most studies have revealed that AAT, particularly dog therapy, has a “calming effect” on patients with dementia and Alzheimer’s disease [14,15,16,17]. This bond can be helpful as a communication link during therapy sessions and can decrease agitation behaviors.

Cerebral palsy (CP) is a non-progressive neurological condition that results in limitations in motor function. Children with CP often require multiple health, rehabilitation, and community health care services [18]. Almasri *et al.* examined the child, family and service characteristics of families that include children with CP. They found that limited child gross motor function was a risk factor but that the perception of family-centered services was a protective factor. Health care providers need to understand this evidence and thereby provide better quality services for their patients [18].

It is well known that the incidence of aggression, agitation, social withdrawal, depression, and psychotic disorders is growing in children with cerebral palsy and physical and mental disabilities. These disorders are observed in rehabilitation centers and special care units and by staff and family members of patients. Furthermore, environmental factors in rehabilitation centers and other health care units have become barriers for therapy. Under these conditions, AAT and other animal activities may be helpful in coping with these difficulties by presenting a different method and a more humanistic therapy environment.

AAT aims to benefit from animal companionship during a targeted therapy, to achieve optimal results in patients, and to support the therapy. It provides positive effects, such as adapting to stressful situations and hospital environments; decreasing anxiety, stress, pain and blood pressure; and increasing mobility and muscle activity. Guiding animals increase physical activity, help prevent mental states such as loneliness and depression, improve daily life activities and provide social support by increasing quality of life [19,20,21,22,23,24].

### Why Do We Need a New Therapy Model for the Rehabilitation of Children with CP and Other Disabilities?

Physical therapy (PT) plays a central role in managing CP; it focuses on function, movement, and the optimal use of a child's potential. Physical therapists use physical approaches to promote, maintain and restore the physical, psychological and social well-being of disabled people. PT interventions are preferred increasingly by therapists, doctors and parents [25]. However, we need to understand the effects of PT interventions for evidence-based decision-making. AAT is a widely used PT intervention that is currently supported by limited evidence. Substantial research has investigated the physical and social effects of equine-assisted therapies to improve the well-being (social, cognitive, psychosocial, and physical) of individuals with CP [26,27]. Equine-assisted therapy studies have indicated that parents of children with CP were satisfied in categories such as connection, developmental gains, socioemotional gains, and personal gains [26,27]. However, there is a gap in showing the synergistic effect of AAT and occupational therapy goals in children with cerebral palsy and physical and mental disabilities. The current study aimed to evaluate the effects of dog-assisted therapy on occupational therapy needs in the rehabilitation of children with cerebral palsy and physical and mental disabilities.

## 2. Methods

This qualitative study was conducted in a private training and rehabilitation center in Antalya under the supervision of an experienced team between 2008 and 2011. A total of 10 patients with various disabilities were enrolled in the study. We used the mechanism of action of AAT, as identified by Ballarini [4] and The Lifestyle Performance Model developed by Velde and Fidler [28], to evaluate the effects of occupational therapy in our patients. In the current study, therapy sessions with five study groups were recorded by a therapist using videotaping and photography. We used qualitative techniques, including interviews and observation, for study evaluation.

### 2.1. Materials

#### 2.1.1. AAT Mechanism of Action

**Figure 1:**
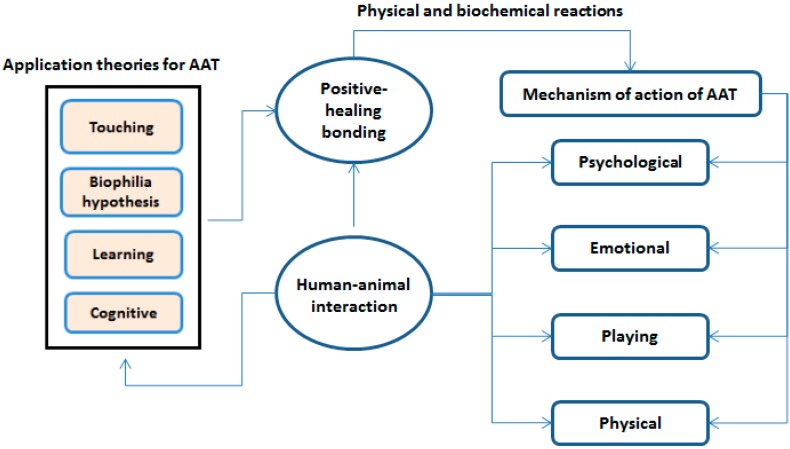
Mechanism of action of AAT and theories for the therapies.

The mechanism of action of AAT is based on positive-healing bonding, which occurs in human-animal interactions, and psychological, emotional, playing and physical mechanisms cause physical and biochemical reactions by activating this bond [4,29]. The primary structures that activate these mechanisms in patients should be structured according to four theories: touching, biophilia, learning and cognitive theories [30]. In animal-assisted therapy, all types of applications, which are performed according to these four theories, can provide various benefits (Figure 1).

#### 2.1.2. Occupational Therapy and the Lifestyle Performance Model

Occupational therapists believe that AAT can transfer the internal responses of the patient to the external environment. During the AAT process, purposeful activities can be useful for gaining skills or actions that satisfy personal needs [31]. For example, if a patient enjoys doing something such as gardening, the occupational therapist could include gardening in therapy to help the patient recover their impaired body functions or skills. Including a purposeful activity in the occupational therapy maximizes the patient’s benefits from therapy [31,32]. We used a purposeful activity for each patient in addition to a therapy dog during occupational and physical therapy in the current study.

Velde *et al.* revealed that AAT is a suitable method for occupational therapy and stated that The Lifestyle Performance Model can be used for the analysis and interpretation of positive outcomes of animal-assisted therapy in an occupational therapy context [31]. The Lifestyle Performance Model consists of five domains that are compatible with AAT. These domains are intrinsic gratification, societal contribution, reciprocal interpersonal relationships, self-care and self-maintenance and responsive environment. The first four domains are related to human performance. A responsive environment facilitates pet-related activities [28].

### 2.2. Study Design

We established five study groups with therapy dogs according to the therapists’ goals and patient needs to evaluate the effect of AAT on the rehabilitation and wellbeing of children with various physical and mental disabilities (Table 1). Three different dogs were used in the therapy process.

**Table 1:** Characteristics of study participants.

No of Study Group	Age of Patients	Number of Patients	Gender	Disability Type
I	9, 14, 23	3	2 male and one female	Mental and physical disabilities
II	4	1	Male	Cerebral palsy and hemiparesis
III	5	1	Male	Bilateral cerebral palsy
IV	22, 11, 8, 18	4	2 male and 2 female	Mental and physical disabilities
V	5	1	Male	Spastic type of cerebral palsy

We identified the occupational needs of each patient through an interview with their parents and observed their physical and social disabilities. We developed each study group to improve the occupational needs of patients based on The Lifestyle Performance Model and the mechanism of action of AAT. We included each patient’s occupational needs, the therapists’ goals, and a dog-assisted therapy, consistent with The Lifestyle Performance Model (Table 2).

**Table 2:** Descriptions of study groups.

No of Study Group	Patients’ Occupational Needs	Activity with Dog-Assisted Therapy	The Lifestyle Performance Model	Expected Therapy Results
I	Reduction in fear from medical interventions, anxiety and stress in the hospital environment	Health control activities with therapy dog. These activities were planned to trigger the touching, learning and cognitive mechanisms of AAT	Internal emotional gratification, Social contribution, Reciprocal relationship	Improving skills of children, Increasing self-confidence, Decreasing fear and anxiety
II	Use the right side of his body, Increase active motility on the same side	Giving cold yogurt to therapy dog using the child’s foot to create a vibration from sequentially warm tongue of the dog, Feeding the dog and playing football with the dog to do active movements.	Intrinsic gratification, Self-care and self-maintenance, Reciprocal relationship, Responsive environment	Increasing motor learning with high motivation, Relaxation of general body, Decreasing body tonus and gaining a general feeling of comfort
III	Increasing muscle activities (hand skills, standing up in balance)	Writing, painting, Feeding therapy dog	Internal emotional gratification, Social contribution, Reciprocal relationship, Responsive environment	Increasing the motivation of the child, Improving body balance and skills requiring small muscle movements
IV	Increasing communication, planning and empathy skills	Birthday celebration activities (baking a cake and organizing a party) with the therapy dog	Societal contribution, Reciprocal interpersonal relationships, Responsive environment, Self-care and self-maintenance	The goal of the therapist was to improve the empathy skills of children with people and living creatures around them, communication skills; help gain team spirit and the skill of being a team member and provide a feeling of belonging
V	Decreasing the risk of contracture development, Increasing mobility to prevent constipation	Relaxation studies for muscle tonus in lower and upper extremities with the therapy dog	Responsive environment, Self-care and self-maintenance, Societal contribution	Regulating muscle tonus and preventing contracture, Increasing active movements

### 2.3. Ethics

The study was planned and performed after the children’s physicians confirmed that these activities were safe; the patients were then directed to the rehabilitation center. The entire study process was explained to the parents and children. Written signed consent was obtained from the parents who confirmed that their child could participate in this study. The present study followed the principles of the Declaration of Helsinki. The therapy dogs’ health states and therapy compliance were confirmed, and they were approved by veterinary surgeons and AAT experts.

## 3. Results

### 3.1. Group I

Two male subjects and one female subject, who had different disabilities and were aged 9, 14, or 23 years, underwent dog-assisted therapy in March 2011. Special children are exposed to many different medical interventions beginning in infancy and continuing throughout their lives. Therefore, simple medical controls such as vaccinations and going to the doctor’s office may frighten them, and they become anxious. Being in the stressful hospital environment can negatively affect the children themselves, their family members, and healthcare personnel.

**Figure 2:**
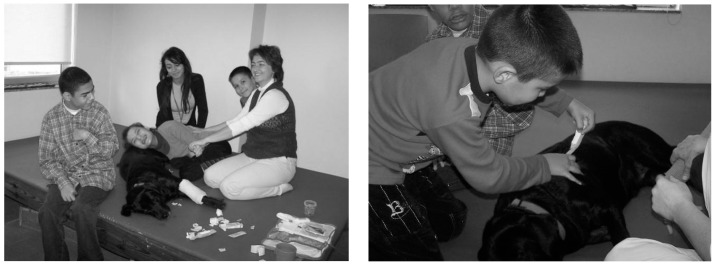
Dog-assisted therapies and activities in children of various ages and mental and physical disabilities who were frightened by health checks.

The goal of the children in this group was to prepare Komur (therapy dog) for the health check that it would attend shortly. The goal of the therapist was to improve the children’s ability to cope with their own anxieties and fears; to define a goal; to plan; and to improve their skills to implement the plan. During these practices, children could improve their skills to use their own bodies according to their capabilities; to develop empathy with themselves and with a different living being; to improve their skills of receiving and presenting help; and to develop their communication skills (Figure 2).

The children knew that Komur was frightened by the veterinary surgeon. They thought that if they played this game with their friend (Komur), they would encourage Komur and the dog would be less frightened when it had to go to the health check. They applied many simple examinations on Komur. They used toys and real materials that were present in Komur’s health bag. They measured the dog’s temperature, listened to its abdomen, listened to its lungs using a stethoscope, performed vaccination by using an injector, and bandaged its feet by using surgical dressings. At the end of the study, the children thought that they had achieved their goals. Additionally, the therapist achieved the primary goals of the group according to the various needs of each child.

### 3.2. Group II

The second study group included a 4-year-old boy who had cerebral palsy and hemiparesis, and the therapy was performed in July 2004. Applications to use the right side of his body were performed with the therapy dog. He had difficulty in using the right side of his body. Muscle tonus was also increased on the same side. He was faced with muscle shortening and deformity formation risks in the lower and upper extremities, where the muscle tonus was high. Moreover, he preferred to not use the right side of his body. His awareness of the right side of his body was quite low. He was performing actions by using only the left side of his body while moving and defining movement plans.

**Figure 3:**
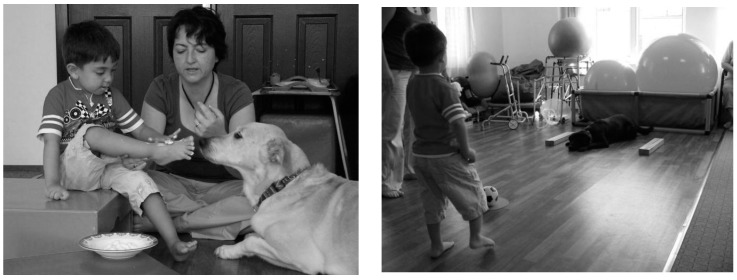
Dog-assisted therapies and activities in a child with cerebral palsy and hemiparesis.

In the present study, the goal of the therapist was to help increase the muscle tonus regulation by providing various sensorial stimulations on the right side of his body and to increase the active motility of that side. Preventing contracture and providing coordination between the hemispheres of the brain were additional goals. The goal of the child was to give his dog yogurt and enjoy the experience. Cold yogurt followed by the warm tongue of the dog, as well as the vibration triggered by the dog’s licking, aided the decreased muscle tonus and increased the awareness of that extremity (Figure 3).

Increasing the circulation to that part of the body provided warming for the extremity. By causing the extremity to relax, the general body tonus started to decrease, and a general feeling of comfort was gained. However, the decreased body tonus and the feeling of comfort were temporary. Therefore, performing active movements immediately after this gain was quite important, and these movements eased motor learning. A child who would like to feed or play football with a dog should start to perform the active movements repeatedly. Therefore, the repetition requirement for motor learning is developed spontaneously, without forcing and with high motivation.

### 3.3. Group III

**Figure 4:**
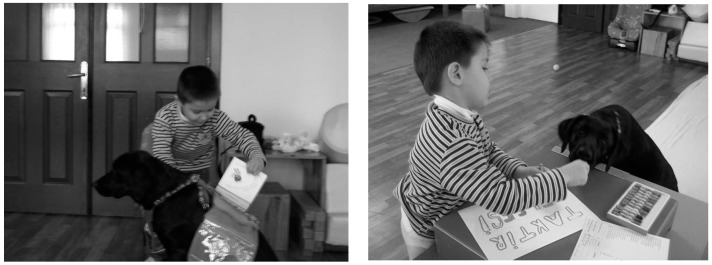
Dog-assisted therapies and activities in a child with bilateral cerebral palsy.

In the third group, a 5-year-old boy participated in therapy in February 2008. The boy had bilateral cerebral palsy, and his right side was affected more than his left. Muscle activities such as hand movements and balanced standing were performed with the therapy dog. He received a good training report because he came to the center for the entire year and performed his homework. He received his training report and a testimonial that could be painted from his beloved friend Komur, who helped him while he was studying, as shown in Figure 4.

The boy was delighted with his report and showed the dog how well he could paint. The boy also intermittently gave some of the pretzels that he was eating to Komur. In this group, the goal of the therapist was to increase the motivation of the child, who was trying to perform activities that challenged him both physically and mentally due to his small age and to provide continuity of therapy by facilitating the acceptance of future studies. During the activities, the goal was to stand with balance, and the boy improved the skills that required small muscle movements while he was standing.

### 3.4. Group IV

The fourth group included a 22-year-old girl, an 11-year-old blind girl, and 8- and 18-year-old boys with various mental and physical characteristics (one of them had both severe physical disability and blindness); the therapy was conducted in September 2012. The therapy dog (Joey) and its friends were included in this group. Various activities were performed to improve communication, planning and empathy skills with the therapy dog. Birthday celebration activities were performed. The goal of the children was to bake a cake and organize a party for Joey. For this purpose, they planned to invite people whom Joey loved and its dog friends to the party.

Joey was a 9-year-old male golden Labrador half-breed who was born in the USA and had been to Israel, Istanbul, and Antalya. The dog initially guarded the therapy center, but it was later trained to become a therapy dog after its veterinarian watched it perform rehabilitation activities. According to the physical therapist, Joey was a clever therapy dog. After the dog had finished training, it started to work with physical therapist, accompanied by his vet. The physical therapist said that the dog had changed their lives at the center as well as the lives of children and their parents who came to therapy.

The goal of the therapist was to improve the children’s empathy skills with people and living creatures around them and communication skills and to help them gain team spirit, the skill of being a team member, and a feeling of belonging. As the dog helped the children experience various smells, textures, and consistencies in a sensorial fashion, the children could improve their skills to use their bodies according to their physical capabilities and to improve their physical and mental coordination, motor planning skills, goal definition and goal-oriented planning skills. Therefore, their effort would result in a product and a successful process.

**Figure 5:**
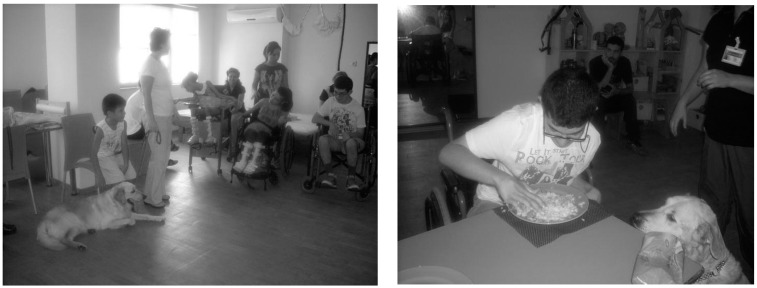
Dog-assisted therapies and activities in children with mental and physical disabilities.

The children baked a birthday cake for Joey, Komur and its friend visitor dog, Gaia. They brought all of the ingredients for the cake themselves. While using the ingredients, they smelled, touched, kneaded, and talked about them. They planned how to prepare the cake, and they blew out the candles on this cake for Joey and its friends and cut the cake. Then, they sang a happy birthday song for them; they danced while listening to music, and as a result, they achieved their goals (Figure 5).

The dogs also achieved their goals, and they ate a cake with fish and rice. The therapist achieved her goals, which she had pre-specified for each child separately according to their needs. Other people were invited and participated in the birthday party. During the therapy sessions, the dog is required to not make contact with or play with the child all of the time. By relying only on its presence, many activities with pre-specified goals and achievements can be planned. The children continued to feel Joey’s presence after its death, and they wrote letters, drew pictures for Joey, and tried to continue to live with it.

### 3.5. Group V

**Figure 6:**
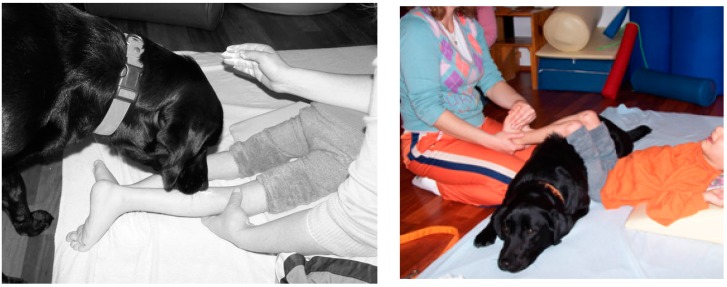
Relaxation practices in a child with spastic type of cerebral palsy accompanied by the therapy dog.

The fifth group included a 5-year-old boy with the spastic type of cerebral palsy, which affected him bilaterally. He was a child with intense muscle tonus in his upper and lower extremities, he had limited active movements, and he was at high risk for contracture development. Because he was inactive, he had frequent constipation issues. Relaxation studies were performed for muscle tonus in his lower and upper extremities with the therapy dog. In this group, the goal of the child was to give lunch to his friend Komur by using his hands and feet and to put Komur to sleep by lying down with it, as shown in Figure 6.

The boy thought that Komur had not yet had its lunch nap. The goal of the therapist was to help the boy regulate his muscle tonus and thereby prevent contracture development. Active movements of his body were increased, and his bowel motility regulation was supported by using the body temperature and body oscillations of the dog.

## 4. Discussion

In the present study, the groups underwent therapy activities, and the observed changes in children were recorded during therapy. These therapy groups were the first to scientifically apply dog-assisted therapy under the supervision of an experienced team. We found consistent results with the literature. The changes observed at baseline, during and after therapies were similar to those observed in previous studies [33,34]. Barol observed and recorded processes in an autistic male child after applying dog-assisted therapy [34]. Before the application, the therapists and the investigator held a meeting and discussed which dog therapy activities would provide motivation, which included improving hand-skills, awareness, self-esteem, and behaviors such as playing with a ball, cutting with scissors, feeding (serving), tracking, following, bringing water to the dog, and brushing the dog. At the end of therapy, improvements or disappearances of temper tantrums; improvements in focusing on something by playing games, in selection ability, in independent behaviors, in decision making, in attention; increases in awareness, communication desire, being sensitive of surroundings, being happy, and improvements in behaviors and moods were observed [34]. In our study, similar improvements were also found in children with cerebral palsy during the treatment processes.

Dog-assisted therapies and the activities conducted on patient groups have substantially facilitated treatment compliance, particularly in children [35,36]. Previous studies investigating various health problems have shown that animal-assisted therapies and activities may be helpful during routine therapy by achieving optimal results and supporting patient therapy [37,38,39,40,41,42]. Although many studies have examined physiological and motor outcomes in children with cerebral palsy during equine-assisted therapy [43,44,45,46,47], few studies have investigated the performance of dog-assisted therapies and activities in children with cerebral palsy or mental or other physical disabilities [34,35,36]. In the present study, dog-assisted therapies and activities were performed in Turkey, under the supervision of an experienced team, for children with cerebral palsy and physical and mental disabilities. The outcomes were similar to those found in the literature. Our results suggest that dog-assisted activities and therapies can be a supportive method for routine treatment in the rehabilitation of children with cerebral palsy and physical or mental disabilities.

Forbes and Marxen found that occupational therapy with a therapy dog increased the enjoyment of and participation in the therapy process of a child with CP. They observed that a therapist utilized both her own skills and the skills of a dog when the child is motivated by the therapy dog [48]. In our study, visual analysis revealed that occupational and physical therapy with a therapy dog increased children's social adjustment and adaptation to therapy process. Overall, the therapy dog created a more enjoyable, safe and stress-free therapy environment for all individuals, particularly for the patients and therapists who participated in the current study.

Comparative studies of various diseases would provide more detailed information. Studies in this field, which specifically evaluate the effect of animal-assisted therapies on improvements of life quality and the physical, mental and social health of children with physical and mental disabilities or chronic diseases, will provide supportive information and are important to improve rehabilitation [5,6,49]. Additionally, the concept of public health requires individuals to participate in rehabilitation and long-term health care throughout their lifespan to improve their quality of life.

### Limitations

This study has some limitations. *First*, the study is limited to 10 cases in Antalya. We could not compare our results with those a control group that included patients working without a therapy dog. Further studies should assess whether physical and occupational therapy with a therapy dog in children with disabilities has a superior effect compared with other therapy models. *Second*, data collection was limited to interviews and observations. *Third*, the authors of this article had experience using physical therapy and chose to only apply dog-assisted therapy. *Fourth*, the lack of research literature investigating dog-assisted therapy in children with physical and mental disabilities from a physical and occupational therapy perspective did not allow us to compare our findings with other research. Finally, this study was limited by the pre-selection of many participants by the physical therapy center, Private Gercek ILGI Training and Rehabilitation, Antalya. However, if we followed our participants during their daily activities after dog-assisted therapy, our findings could have reflected daily life.

## 5. Conclusions

Despite these limitations, our study findings indicated that children who experienced fear, anxiety and difficulties due to their disabilities in daily life learned to cope with their anxieties and fears and to set goals and create plans to achieve their aims during therapy. Children improved their abilities to use their bodies according to their capabilities. Accordingly, they improved their skills in developing empathy between themselves and a therapy dog, receiving and presenting help, and improving communication skills. The results of the present study revealed that dog-assisted therapies and activities can be a supportive method for routine treatment in the rehabilitation of children with cerebral palsy and physical and mental disabilities.

In conclusion, the individual and social benefits gained by dog-assisted therapy may aid in the prevention, improvement and development of children with various disabilities. Dog-assisted therapy can be developed and used as a supportive tool in veterinary public health to help the rehabilitation of children with disabilities.
